# Supplementary material for: GcvB Regulon Revealed by Transcriptomic and Proteomic Analysis in Vibrio alginolyticus
Source: Int J Mol Sci. 2022 Aug 20;23(16):9399. doi: 10.3390/ijms23169399 (PMC9409037; doi:10.3390/ijms23169399)
Supplement: Supplementary file 1 [file ijms-23-09399-s001.zip › ijms-1845653-SI.pdf]

Table S1 Statistics of the transcriptome sequencing data

Group	Sample	Clean Reads	HQ Clean Reads	Q20 (%)	Q30 (%)	GC Content (%)	Reads mapped to genome (%)
ZJ-T	1	25602240	25389872	98.30	94.77	46.58	95.04%
	2	21682388	21541760	98.52	95.22	46.90	96.42%
	3	16938122	16807610	98.47	95.11	46.88	96.51%
ZJ-T-△ *gcvB*	1	21670674	21521282	98.46	95.10	46.99	96.44%
	2	17402972	17260668	98.45	95.08	46.74	96.40%
	3	25841482	25552878	98.47	95.12	47.03	96.03%

Table S2 Nucleotide sequences of primers used in this study

Primers	Sequence (5'-3')
For *gcvB* mutant strain construction	
pSW7848_F	GTCTGATTCGTTACCAATTATGACAAC
pSW7848_R	GAATTCGATATCAAGCTTATCGATAC
gcvB-UP_F	ataagcttgatatcgaattcTAAGCAGGTTTCTCACTAC
gcvB-UP_R	tatttcacccTCTATCAAAAATCTCATCCG
g*cvB*-DOWN_F	ttttgatagaGGGTGAAATATAGAATTTATCTG
gcvB-DOWN_R	taattggtaacgaatcagacAGTGATTCATCCAATACTTC
Del-check-pSW7848-F	TCACTGTCCCTTATTCGCACC
Del-check-pSW7848-R	CTGCTTTTGAGCACTACCCG
Δ-*gcvB*-F	AGTATCCTTGAGCAAGTGGTTC
Δ-*gcvB*-R	CTACGTTCCAACACCTTATCTGTC
For *gcvB* complementary strain construction	
pMMB207_F	AGAAGCGGTCTGATAAAACAGAATTTGC
pMMB207_R	GCGCAACGCAATTAATGTAAGTTAG
gcvB-F	ttacattaattgcgttgcgcAAATACGTTAAAAGGCGCAAATTG
gcvB-R	tgttttatcagaccgcttctTGTCCGTTGTCGAACAGC
pMMB207-check-F	ATGCTCTTCTGCTCCCGAAC
pMMB207-check-R	ACGGCGTTTCACTTCTGAGT
For translational fusions	
pSCT32-gfp_F	GTGAGCAAGGGCGAGGAGCT
pSCT32-gfp_R	TAAATGCATGCCGCTTCGCCTT
pSCT32-gfp-check-F	CGCTATCGCTACGTGACTGG
pSCT32-gfp-check-R	GTTTCACTTCTGAGTTCGGCATG
cysK-TL_fwd	GGCGAAGCGGCATGCATTTAAAGACGTCATTTGTCAGG
cysK-TL_rev	AGCTCCTCGCCCTTGCTCACGTAGATTTTGCTCATAGTGATG
cysN-TL_fwd	GGCGAAGCGGCATGCATTTACCCTTGGTTGTTATCCATTG
cysN-TL_rev	GCCAAGCTTGGTACCAGATCAACTGCACTGTTCATTATC
ilvG-TL_fwd	GGCGAAGCGGCATGCATTTACCTACTAAGGTAAACCCTGG
ilvG-TL_rev	AGCTCCTCGCCCTTGCTCACTTGTGCACCAGTCATCGTAAAC
For T7 in *vitro* transcription	
T7-*gcvB*-F	TAATACGACTCACTATAGGGCGCAACACGGGCCTGAAC
T7-*gcvB*-R	TAAAAAAACACCGCCTAATTTAGGCG
For Hfq recombinant protein construction	
pET28b_F	CACCACCACCACCACCAC
pET28b_R	GGTATATCTCCTTCTTAAAGTTAAACAAAATTATTTC
Hfq-ORF_fwd	tttaagaaggagatataccATGGCTAAGGGGCAATCTC
Hfq-ORF_rev	tcagtggtggtggtggtggtgATCTTCAGATTTCTCTTGTGGAC
pET28b-check-F	aagtggcgagcccgatcttc
pET28b-check-R	CTAGGGCGCTGGCAAGTGTA

For qPCR	
q-*gcvB*-F	CAGGGAGTGTGGACAAAACA
q-*gcvB*-R	TGAACGGCTTGATTTCTTTGGA
q-*ilvC*-F	ACCGGTCACTTCTCTAGCAC
q-*ilvC*-R	GAATGCTAGCTCAACACCCG
q-*ilvD*-F	TGCAACGGGTGTGAAAGAAG
q-*ilvD*-R	TTCTTTCGCAATACCGCCAG
q-*ilvE*-F	CACTGCGCCAAAACAAACTC
q-*ilvE*-R	TCCAGCTTGAAATCATCGCG
q-*cysM*-F	ACGACACTAAGCTTTGCGTG
q-*cysM*-R	CCGACTTTACGCCCAATCAG
q-*cysK*-F	ATGAGCCTAGAACGCCGTAA
q-*cysK*-R	CGTCGATTTCACCGTCTGTC
q-*cysD*-F	GGGCTGTAGTCCGTTTGTTC
q-*cysD*-R	TGAATACACGCGCTCTTTCG
q-*cysN*-F	AAGCACTTTGTTGTCGCGAT
q-*cysN*-R	ATCCACCACATTGTCCCCTT
q-*cysH*-F	TAGTTACTCGCGCCAAAACG
q-*cysH*-R	TCAATTCCCTCTACGCCCTG
q-*cysI*-F	GCTTAATGGATGACGGCGTT
q-*cysI*-R	GACACCCAGTTACACGCAAA
q-*cysJ*-F	TTGGTTTGCTTCGTCGTCTG
q-*cysJ*-R	TCTTCGAGACGCTGTGCTAA
q-*hutU*-F	TAAACGCGCTACTCAACACG
q-*hutU*-R	ATGACGCATGACACCAGTTG
q-*hutG*-F	TGGCGTCTCATTACTCGGTT
q-*hutG*-R	GCTTGAAATGGTTGCTGCAC
q-*hutI*-F	GCACGCTATTTCGCCTTACA
q-*hutI*-R	CTACCAGACGTTTTGCTCCG
q-16S-F	CTGGAACTGAGACACGGTCC
q-16S-R	CTCGCACCCTCCGTATTACC

Table S3 *Vibrio alginolyticus* genes and/or proteins are up/down regulated significantly in *gcvB* knockout strain

id	Symbol	Description	T-FC	T-FDR	P-FC	P-FDR
**Metabolism**						
BAU10_18365	*VC_1589*	alpha-acetolactate decarboxylase	-2.29	0.0107	/	/
BAU10_00850	*mtlD*	mannitol-1-phosphate 5-dehydrogenase	5.72	0.0000	2.48	0.0003
BAU10_00855	*mtlA*	PTS mannitol transporter subunit II	2.47	0.0429	1.82	0.0171
BAU10_21550	*otnI*	hydroxypyruvate isomerase family protein	/	/	1.52	0.0001
BAU10_18845	--	NADH:ubiquinone oxidoreductase	-2.76	0.0225	/	/
BAU10_20290	*prkC*	serine/threonine protein kinase	-2.04	0.0207	/	/
BAU10_13125	*argF*	Ornithine carbamoyltransferase	4.58	0.0147	/	/
BAU10_13135	*pyrB*	aspartate carbamoyltransferase catalytic subunit	-2.69	0.0044	/	/
BAU10_13665	*argB*	acetylglutamate kinase	6.70	0.0347	/	/
BAU10_13670	*argC*	N-acetyl-gamma-glutamyl-phosphate reductase	5.66	0.0107	/	/
BAU10_09145	*metE*	methionine synthase	-3.14	0.0004	/	/
BAU10_05660	*hutU*	urocanate hydratase	2.14	0.0083	/	/
BAU10_05665	*hutG*	formimidoylglutamase	2.72	0.0000	/	/
BAU10_05670	*hutI*	imidazolonepropionase	2.32	0.0024	/	/
BAU10_03770	*cysM*	cysteine synthase/cystathionine beta-synthase family protein	5.56	0.0007	-1.68	0.0097
BAU10_03130	--	putative nitrogen regulatory protein P-II family protein	2.80	0.0207	/	/
BAU10_00735	*leuB*	3-isopropylmalate dehydrogenase	/	/	1.99	0.0000
BAU10_13495	*cysI*	assimilatory sulfite reductase (NADPH) hemoprotein subunit	/	/	-2.36	0.0000
BAU10_15025	*ilvG*	acetolactate synthase II large subunit	/	/	3.86	0.0000
BAU10_13500	*cysJ*	sulfite reductase [NADPH] flavoprotein, alpha-component	/	/	-2.43	0.0000
BAU10_13490	*cysH*	phosphoadenosine phosphosulfate reductase	/	/	-2.30	0.0000
BAU10_22370	*tdh*	L-threonine 3-dehydrogenase	/	/	2.16	0.0000

BAU10_00495	*cysN*	sulfate adenylyltransferase subunit CysN	/	/	-2.27	0.0000
BAU10_15045	*ilvA*	threonine ammonia-lyase, biosynthetic	/	/	1.79	0.0000
BAU10_00490	*cysD*	sulfate adenylyltransferase subunit 2	/	/	-2.36	0.0001
BAU10_00730	*leuC*	3-isopropylmalate dehydratase large subunit	/	/	2.17	0.0003
BAU10_22375	*kbl*	glycine C-acetyltransferase	/	/	2.07	0.0000
BAU10_00725	*leuD*	3-isopropylmalate dehydratase small subunit	/	/	2.26	0.0000
BAU10_17915	*aroG*	3-deoxy-7-phosphoheptulonate synthase AroG	/	/	1.81	0.0000
BAU10_17355	*OCC_04335*	aminotransferase, classes I and II superfamily	/	/	2.01	0.0000
BAU10_23920	--	lactoylglutathione lyase	/	/	1.59	0.0443
BAU10_05890	--	peptidase S41	/	/	-1.75	0.0316
BAU10_22890	*MGYG_04702*	putative carboxypeptidase G2	/	/	-1.53	0.0006
BAU10_17520	*dgkA*	diacylglycerol kinase	3.80	0.0153	/	/
BAU10_09900	--	aryl-sulfate sulfotransferase	3.15	0.0083	/	/
BAU10_18320	*HI_0002*	putative long-chain-fatty-acid-CoA ligase	/	/	1.51	0.0000
BAU10_11015	*glpF*	glycerol uptake facilitator protein GlpF	/	/	1.60	0.0160
**Transport**						
BAU10_20535	*artP*	arginine ABC transporter ATP-binding protein ArtP	5.52	0.0249	/	/
BAU10_17740	*mdtN*	HlyD family secretion protein	2.21	0.0327	/	/
BAU10_07410	*aapJ*	amino acid ABC transporter, periplasmic amino acid-binding protein	/	/	2.69	0.0000
BAU10_15175	*patH*	amino acid ABC transporter, periplasmic amino acid-binding portion	/	/	-1.59	0.0000
BAU10_02555	*metN*	methionine ABC transporter ATP-binding protein MetN	/	/	1.57	0.0001
BAU10_20240	*tssC*	type VI secretion system contractile sheath large subunit	-2.06	0.0072	/	/
BAU10_20245	*tssB*	type VI secretion system contractile sheath small subunit	-2.09	0.0078	/	/
BAU10_20285	*tagH*	type VI secretion system-associated FHA domain protein	-2.81	0.0083	/	/
BAU10_07935	*yscB*	type III secretion system chaperone	/	/	1.99	0.0000
BAU10_07880	--	type III chaperone	/	/	2.44	0.0002

BAU10_07820	*yscP*	type III secretion system needle length determinant	/	/	1.94	0.0017
BAU10_07915	*yscF*	type III export protein YscF	/	/	2.22	0.0038
BAU10_07960	*exsE2*	Type III secretion regulator	/	/	2.30	0.0457
BAU10_17790	*secF*	putative protein-export membrane protein	/	/	1.94	0.0316
BAU10_07780	*yscV*	type III secretion protein V	/	/	2.75	0.0282
BAU10_22965	*sstT*	sodium/dicarboxylate symporter	/	/	4.70	0.0000
BAU10_18025	*iutA*	TonB-dependent receptor	/	/	1.59	0.0000
BAU10_07860	--	cation transporter	/	/	2.92	0.0316
BAU10_19990	*dcuB*	anaerobic C4-dicarboxylate transporter	/	/	-1.92	0.0020
**Cellular process**						
BAU10_17000	*ompV*	MipA/OmpV family protein	3.00	0.0042	/	/
BAU10_22900	*yjiH*	membrane protein	/	/	-1.88	0.0017
BAU10_09875	*bdlA*	methyl-accepting chemotaxis protein	/	/	1.69	0.0000
BAU10_21265	*pctA*	methyl-accepting chemotaxis protein	/	/	3.27	0.0001
BAU10_19335	*tcpI*	methyl-accepting chemotaxis protein	/	/	-1.52	0.0078
**Genetic information processing**						
BAU10_16040	*VP0184*	DNA repair protein RadC	2.18	0.0068	/	/
BAU10_14995	--	ribonuclease H	3.19	0.0074	/	/
BAU10_00990	--	HNH endonuclease	2.00	0.0251	/	/
BAU10_04605	*ssb*	Single-stranded DNA-binding protein	1986.67	0.0327	/	/
BAU10_20810	*SAR0107*	putative AraC-type regulatory protein	-2.03	0.0078	/	/
BAU10_13685	--	predicted transcriptional regulator	2.19	0.0005	/	/
BAU10_08050	*rbcR*	LysR family transcriptional regulator	/	/	4.63	0.0000
BAU10_14150	*groL1*	chaperonin GroEL	-2.10	0.0007	/	/
BAU10_14155	*groS1*	10 kDa chaperonin GroES protein	-2.86	0.0078	/	/
**Unknown function and hypothetical protein**						

BAU10_21320	--	hypothetical protein	4.31	0.0000	/	/
BAU10_17525	--	hypothetical protein	3.23	0.0182	/	/
BAU10_00805	--	DUF3316 domain-containing protein	2.62	0.0424	/	/
BAU10_07600	--	DUF3316 domain-containing protein	2.27	0.0300	/	/
BAU10_08055	--	hypothetical protein	5.70	0.0016	/	/
BAU10_10975	--	TDT family transporter	2.30	0.0078	/	/
BAU10_13100	--	DUF2061 domain-containing protein	-2.65	0.0015	/	/
BAU10_09150	--	DUF1852 domain-containing protein	-4.28	0.0000	/	/
BAU10_06490	*HI_1246*	LTA synthase family protein	2.67	0.0207	4.01	0.0006
BAU10_21320	--	hypothetical protein	/	/	7.72	0.0003
BAU10_08435	--	hypothetical protein	/	/	-1.74	0.0004
BAU10_07875	*vopS*	Va1686	/	/	2.52	0.0000
BAU10_07920	--	VseE	/	/	1.89	0.0000
BAU10_06625	*Ping_1243*	heme-degrading domain-containing protein	/	/	1.69	0.0029
BAU10_18215	--	hypothetical protein	/	/	-1.61	0.0145
BAU10_07765	*lcrV*	VcrV	/	/	2.10	0.0043

Table S4 Twenty identified differentially expressed proteins for PPI network construction (STRING score > 700)

protein1	protein2	combined_score
leuD	*leuC*	999
leuD	*leuB*	999
leuD	*ilvG*	982
leuD	*ilvA*	885
leuC	*leuB*	999
leuC	*ilvG*	982
leuC	*ilvA*	889
leuB	*ilvG*	998
leuB	*ilvA*	990
lcrD	*bdlA*	703
bdlA	*tcpI*	995
bdlA	*pctA*	839
tcpI	*pctA*	805
patH	*cysK*	815
cysK	*cysN*	987
cysK	*cysD*	987
cysK	*cysH*	993
cysK	*cysI*	998
cysK	*cysJ*	998
cysN	*cysD*	999
cysN	*cysH*	999
cysN	*cysI*	999
cysN	*cysJ*	999
cysD	*cysH*	988
cysD	*cysI*	999
cysD	*cysJ*	999
cysH	*cysI*	999
cysH	*cysJ*	999
cysI	*cysJ*	999
mtlA	*mtlD*	999
tdh	*kbl*	999
tdh	*ilvA*	900
ilvG	*ilvA*	997
